# Leaf reflectance and functional traits as environmental indicators of urban dust deposition

**DOI:** 10.1186/s12870-021-03308-8

**Published:** 2021-11-13

**Authors:** Jiyou Zhu, Jingliang Xu, Yujuan Cao, Jing Fu, Benling Li, Guangpeng Sun, Xinna Zhang, Chengyang Xu

**Affiliations:** 1 Research Center for Urban Forestry, The Key Laboratory for Silviculture and Conservation of Ministry of Education, Beijing Forestry University, Key Laboratory for Silviculture and Forest Ecosystem of State Forestry and Grassland Administration , Beijing Forestry University, Beijing, 100083 China; 2 Production and Operation Management Department, China Communications Construction Company, Beijing, 100088 China

**Keywords:** Dust deposition, Forecast model, Leaf functional traits, Leaf reflectance

## Abstract

**Background:**

How to quickly predict and evaluate urban dust deposition is the key to the control of urban atmospheric environment. Here, we focus on changes of plant reflectance and plant functional traits due to dust deposition, and develop a prediction model of dust deposition based on these traits.

**Results:**

The results showed that (1) The average dust deposition per unit area of *Ligustrum quihoui* leaves was significantly different among urban environments (street (18.1001 g/m^2^), community (14.5597 g/m^2^) and park (9.7661 g/m^2^)). Among different urban environments, leaf reflectance curves tends to be consistent, but there were significant differences in leaf reflectance values (park (0.052–0.585) > community (0.028–0.477) > street (0.025–0.203)). (2) There were five major reflection peaks and five major absorption valleys. (3) The spectral reflectances before and after dust removal were significantly different (clean leaves > dust-stagnant leaves). 695 ~ 1400 nm was the sensitive range of spectral response. (4) Dust deposition has significant influence on slope and position of red edge. Red edge slope was park > community > street. After dust deposition, the red edge position has obviously “blue shift”. The moving distance of the red edge position increases with the increase of dust deposition. The forecast model of dust deposition amount established by simple ratio index (y = 2.517x + 0.381, *R*^2^ = 0.787, RMSE (root-mean-square error) = 0.187. In the model, y refers to dust retention, x refers to simple ratio index.) has an average accuracy of 99.98%. (5) With the increase of dust deposition, the specific leaf area and chlorophyll content index decreased gradually. The leaf dry matter content, leaf tissue density and leaf thickness increased gradually.

**Conclusion:**

In the dust-polluted environment, *L. quihoui* generally presents a combination of characters with lower specific leaf area, chlorophyll content index, and higher leaf dry matter content, leaf tissue density and leaf thickness. Leaf reflectance spectroscopy and functional traits have been proved to be effective in evaluating the changes of urban dust deposition.

## Background

With the acceleration of urbanization, coal combustion, industrial waste gas and traffic exhaust gas are increasing, and urban environmental pollution problems become increasingly prominent, especially urban air quality problems [1–3]. In recent years, smog and dusty weather have occurred frequently in northern China, and air pollution has become one of the most serious environmental problems in many cities, which attracted extensive attention from many researchers [4]. According to the data of Beijing Environmental Monitoring Center, the primary pollutant in Beijing is inhalable particulate matter. In order to further strengthen the dust control work, the Beijing Municipal Government established an atmospheric coarse particulate matter monitoring network of atmospheric coarse particles covering all streets and towns in the city in 2018 [4, 5]. Dust particulate air pollutants not only seriously poison the respiratory system of urban residents, but also threaten the normal growth of urban vegetation [6]. Studies show that the influence of dust on trees is mainly through dust deposition on leaves and stomatal blockage [4–6]. Dust stagnation on leaf surface will block out 60% of light intensity and reduce photosynthesis of leaves by about 20% [5, 6]. At the same time, dust will also affect the gas exchange between leaves and the outside world, and then affects transpiration, photosynthesis and respiration [5–7]. Therefore, it is urgent to monitor and control air pollution.

Although the current air quality monitoring technology and methods have become increasingly mature, including positioning monitoring stations, multi-dimensional monitoring of unmanned aerial vehicle, vehicle-mounted monitoring and so on [7]. However, the coverage of current monitoring methods is generally small, and there are big time and space limitations. As an important part of the city, urban greening vegetation can not only beautify the landscape, but also improve a series of urban environmental pollution problems to a certain extent. In particular, it plays a vital role in dust prevention and noise reduction, air purification, etc. At the same time, it can also play a sensitive biological indicator role in environmental changes [8]. For example, the monitoring of heavy metals in the air by mosses and ozone by *Malus pumila* mainly involves the response of plants to air pollution and the traditional biological monitoring methods [9, 10]. Therefore, a comprehensive understanding of the ability of urban vegetation to purify the atmosphere and its response to dust pollution is of great significance for urban greening construction and allocation, as well as to deal with dust pollution. Hyperspectrum has many advantages, such as high resolution, abundant information, simple data acquisition, etc. [11–13]. The related research based on forestry hyperspectral mainly focuses on the estimation of plant yield, plant diseases and plant physical and chemical parameters, while the application of plant monitoring in dust pollution is relatively few [14, 15]. In the past, in the process of studying the relationship between leaf dust deposition and spectral characteristics, attention was paid to the changes of spectral characteristics of plant leaves before and after dust deposition, while the comparison of plants in different urban environments was often neglected, and the universal applicability of the prediction model was not verified [16].

During the growth process, plants will form morphological, physiological, phenological and other traits in response to the changes in the external environment during growth process, and to a certain extent, affect the functions of ecosystems [17–20]. Leaves are the basic organs of plant photosynthesis, and their functional traits can better reflect the adaptation strategies formed by plants adapting to environmental changes [21, 22]. The response of plant traits to the environment and adaptation strategies have always been the core issues in ecological research. Plants adapt to environmental changes by adjusting and changing some of their own functional traits, and form different survival strategies such as growth, reproduction and defense [21, 23, 24]. Recent studies show that the relationship between vegetation and environment based on plant functional traits can better reveal the response and adaptation of plants to the environment [25–32]. Generally speaking, the total resources available to plants are limited. If plants invest more resources in one functional trait, they will inevitably reduce the resources investment in other traits, that is, at the expense of the construction and functional maintenance of other traits [17, 33]. The research on plant functional traits mainly focuses on the responses of traits to drought, high temperature, ozone and fertilization [34–37]. At present, there are relatively few studies on plant functional traits by urban atmospheric particulates. As one of the structures with the largest contact area between plants and the external environment, leaves are highly sensitive to their growth environment. It can directly reflect the influence of environment on plants, and is often used to diagnose the relationship between plants and their growing environment. Under the long-term stress of dust pollution, the leaves of plants are covered by dust, which causes their growth to be affected to some extent. Therefore, by analyzing the response of spectral reflectance characteristics of leaves to dust pollution, we can further monitor and predict the environmental quality, and provide theoretical basis for environmental governance decision-making.

Therefore, in order to fully understand the response of leaf hyperspectral characteristics and leaf functional characteristics to urban atmospheric particulate matter, the universal applicability of the constructed prediction model in different urban environments was fully verified. In this study, the common greening tree species (*Ligustrum quihoui* Carr.) in Beijing was taken as the study material, and the leaf samples were collected from three typical urban environments, namely streets, campuses and parks. The spectral reflectance characteristics of *Ligustrum quihoui* leaves before and after dust deposition and under different conditions of dust deposition on leaves were analyzed, and the spectral response characteristics of *L. quihoui* leaves in different urban environments were further verified to be consistent, and the sensitive bands of *L. quihoui* leaves to dust deposition were obtained. Finally, according to the typical spectral parameters, the prediction model of dust deposition on leaves is constructed, and the response of plant functional traits to dust deposition on leaves and its trade-off strategy (allocation mechanism of leaf traits to resources) are explored, which provides theoretical reference for the allocation and selection of urban greening plants and the prediction of dust particle pollution.

## Results

### Amount of dust deposition on leaves in different urban environments

**Fig. 1:**
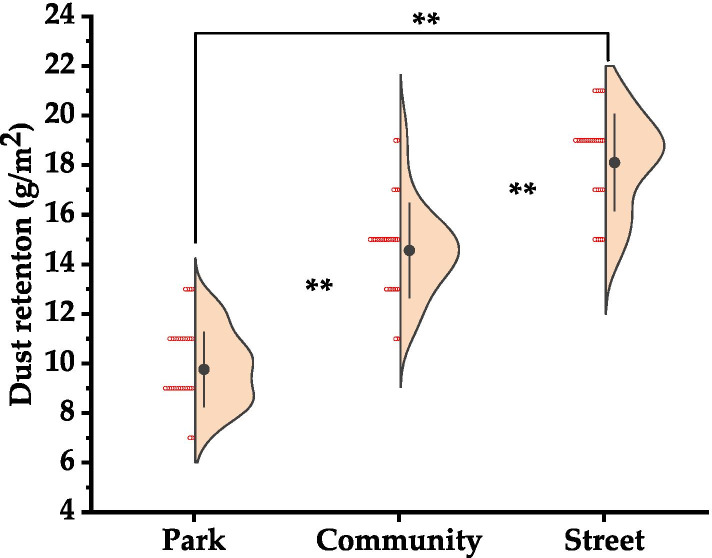
Dust deposition of 180 leaves from the urban tree *L. quihoi* in three urban environments from Beijing, China. “*” indicates that the significant level between treatments is at the *p* < 0.05 level, and “**” indicates that the significant level is reached between the treatments at *p* < 0.01

Streets, communities and parks are typical environments in an urban ecosystem. As the urban dust particles mainly come from automobile exhaust and dust, these three types of environments are divided into relatively obvious dust pollution gradients (streets - high pollution areas, community-moderate pollution areas, parks - relatively clean areas), which are characterized by long-term, stable and real-time. As shown in Fig. 1, the average dust accumulation per unit area of *L. quihoui* leaves was significantly different in different urban environments (*p* < 0.01). The values of dust deposition were streets (18.1001 g/m^2^), communities (14.5597 g/m^2^) and parks (9.7661 g/m^2^).

### Spectral response of *L. quihoui* to dust deposition on leaves

#### Original spectral characteristics

**Fig. 2:**
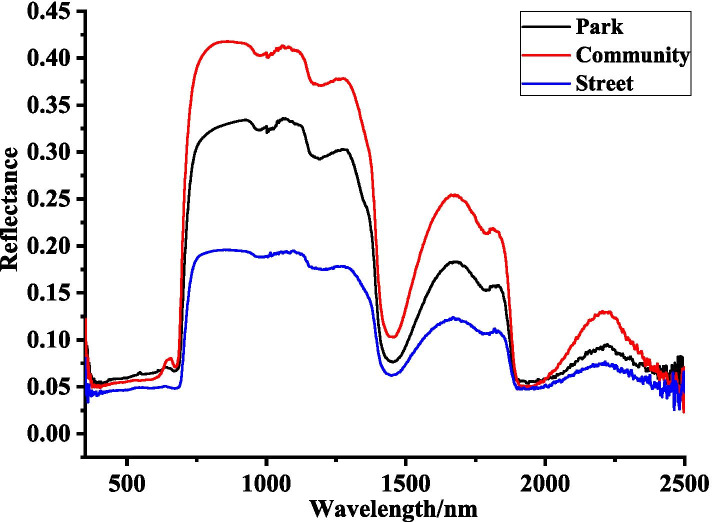
Spectral Signature (350 ~ 2500 nm) of 180 leaves from the urban tree *L. quihoi* in three urban environments from Beijing, China

Leaf reflectance curves (350–2500 nm) significantly varied in three urban environments, from the park (0.052 ~ 0.585), community (0.028 ~ 0.478), and street (0.027 ~ 0.204), thus being negatively associated to the level of dust deposition pollution (Fig. 2). In addition, in the range of 340 ~ 2500 nm, there were five main reflection peaks and five main absorption valleys of *L. quihoui* in three different urban environments, and their positions and ranges were basically the same. The reflection peaks were at 830 nm, 1090 nm, 1276 nm, 1659 nm and 2231 nm, respectively. The absorption valleys were located in the ranges of 360 ~ 678 nm, 940 ~ 1026 nm, 1149 ~ 1231 nm, 1356 ~ 1596 nm and 1885 ~ 2100 nm respectively.

#### Leaf reflectivity characteristics with or without dust stagnation

**Fig. 3:**
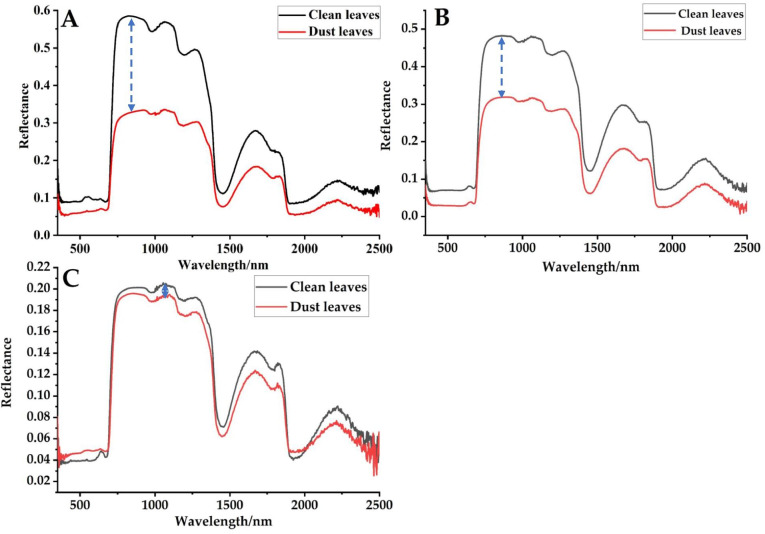
Original leaf reflectance (350 ~ 2500 nm) of 180 leaves from the urban tree *L. quihoi* in three urban environments from Beijing before and after the leaf dust removal. **A** Park, **B** Community, **C** Street

It can be seen from Fig. 3 that the spectral reflectance of dust particles on the leaves of *L. quihoui* was obviously different. In the range of 695 ~ 1400 nm, regardless of the environment, the spectral reflectance of *L. quihoui* with or without dust was the most distinguished, which indicates that this band was the sensitive range of the spectral response. This feature has universal applicability in different environments. In addition, the slope of spectral reflectance curves of plants increased sharply in the range of 686 ~ 780 nm, which is the typical “red edge effect” of plants. However, there was a highly reflective platform in the range of 750 ~ 1310 nm.

#### Spectral characteristics of the first derivative

**Fig. 4:**
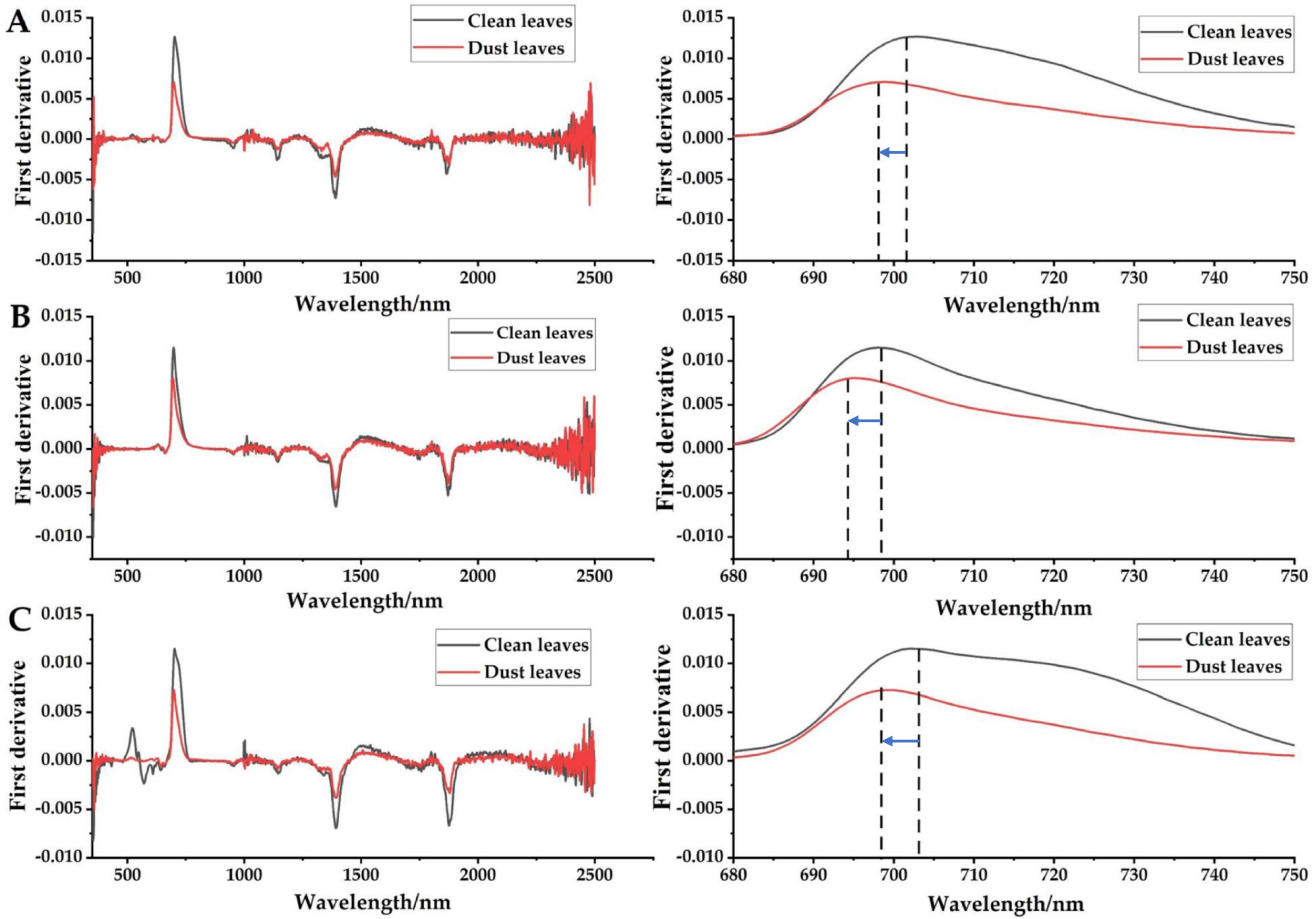
The first derivative spectral curves (350 ~ 2500 nm) of 180 leaves from the urban tree *L. quihoi* in three urban environments from Beijing before and after the leaf dust removal. **A** Park, **B** Community, **C** Street

It can be seen from Fig. 4 that the general trend of the first derivative spectrum (350 ~ 2500 nm) of *L. quihoui* leaves in different urban environments was basically the same, and the dust deposition on leaves has a significant impact on the slope and position of red edge. Among them, in different urban environments, the red edge slopes were parks, communities and streets from large to small. Combined with Fig. 2, it can be seen that the dust deposition amount per unit area in different environments was in the order of park, community and street from large to small, which indicates that *L. quihoui* grows in an environment with high dust concentration, and the greater the dust deposition amount on its leaves, which will directly lead to a sharp decline in the slope of red edge of plant leaf spectrum.

#### Prediction model for dust deposition of leaves

**Fig. 5:**
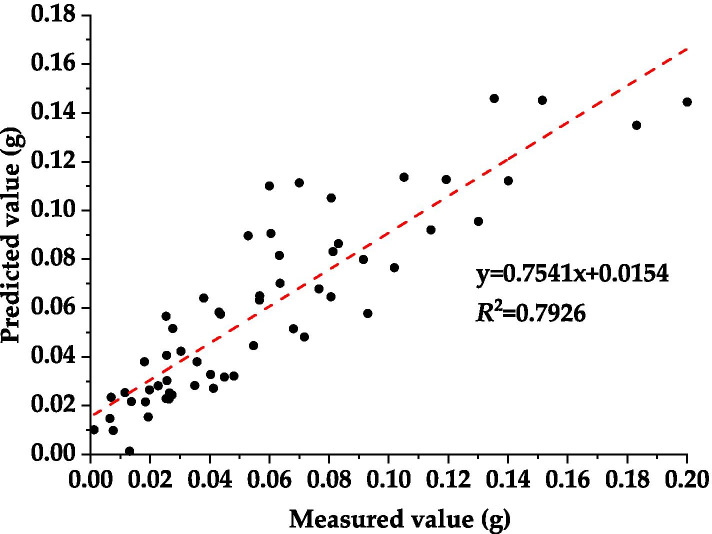
Test of dust deposition prediction model of 180 leaves from the urban tree *L. quihoi* in three urban environments from Beijing based on simple ratio index

Previous studies generally believed that leaf water content index (LWI), red edge index (SDr), normalized index (ND_705_) and simple ratio index (SR) could better characterize the spectral reflectance of plants (Table 1) [38–41]. Therefore, in this study, the spectral parameters were taken as independent variables, and the dust deposition amount per leaf of *L. quihoui* was taken as dependent variable to establish a linear regression model. Sixty leaf samples were randomly selected from each environment to check whether there was a linear relationship between dust deposition and spectral parameters. The stability and accuracy of the prediction model were tested by using the determination coefficient *R*^2^ and root-mean-square error (RMSE). As shown in Table 2, the coefficient of determination *R*^2^ was extremely significant, and the model with simple ratio index (y = 2.517x + 0.381, *R*^2^ = 0.787, RMSE = 0.187) has the best goodness of fit. Finally, we test the model with simple ratio index as independent variable and the predicted value of dust deposition as dependent variable. The results show that the *R*^2^ of the prediction model was 0.793 and the average prediction accuracy was 99.98% (Fig. 5).

**Table 1:** The prediction model of dust deposition based on four typical spectral parameters

Parameter	Definition	Fitting model	***R***^**2**^	RMSE
LWI	R970/R900	y = 2.364x −2.337	0.745^a^	0.202
SD_r_	680 ~ 750 nm sum of first derivative of reflectivity	y = −0.556x + 0.149	0.495^a^	0.224
ND_705_	(R750-R705)/ (R750 + R705 + 2R445)	y = − 0.490x + 0.168	0.658^a^	0.213
SR	R_706_/R_809_	y = 0.313x − 0.107	0.787^a^	0.187

^a^indicates that *R*^2^ of the model reaches a significant level

### Response of plant functional traits to urban atmospheric particulates

**Fig. 6:**
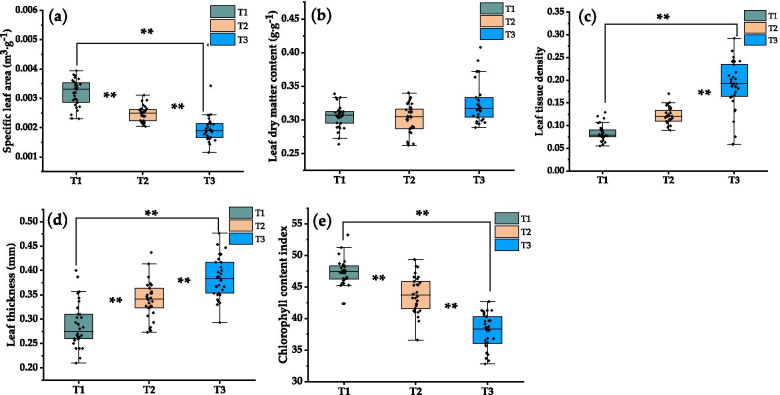
Effect of leaf dust deposition on leaf functional traits of *L. quihoi* in three urban environments from Beijing, China. **a** Specific leaf area, **b** Leaf dry matter content, **c** Leaf tissue density, **d** Leaf thickness, **e** Chlorophyll content index. “*” indicates that the significant level between treatments is at the *p* < 0.05 level, and “**” indicates that the significant level is reached between the treatments at *p* < 0.01

There were significant differences in plant functional traits in different urban environments (Fig. 6). With the increase in dust deposition on the leaf surface, the specific leaf area and chlorophyll content index gradually decreased, and reached extremely significant differences in different environments. Change extremely significant to highly significant (*p* <0.01). On the contrary, leaf dry matter content, leaf tissue density and leaf thickness increased gradually.

### Correlation between plant functional traits and its ecological strategy under the influence of dust deposition

**Fig. 7:**
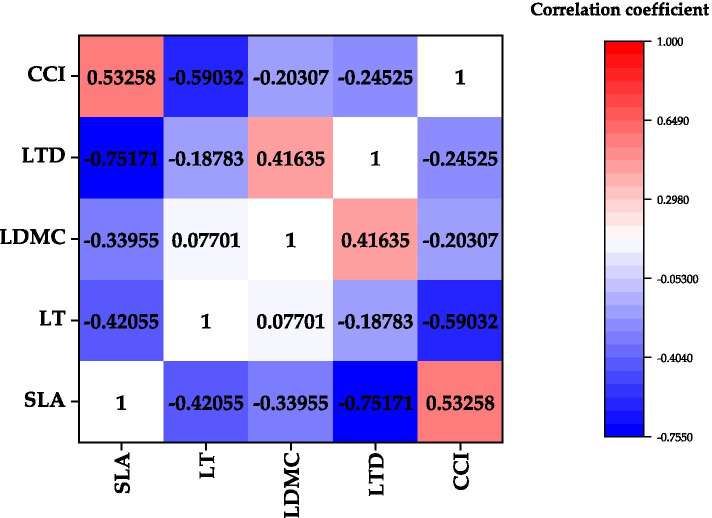
Correlation analysis among plant functional traits of *L. quihoi*. “*” indicates that the significant level between treatments is at the *p* < 0.05 level, and “**” indicates that the significant level is reached between the treatments at *p* < 0.01

Leaf functional traits do not function in isolation, and there was a certain correlation among functional traits (Fig. 7). The specific leaf area was positively correlated with chlorophyll content index. Specific leaf area was negatively correlated with leaf dry matter content, leaf tissue density and leaf thickness. Leaf thickness was negatively correlated with chlorophyll content index. There was a significant positive correlation between dry matter content and tissue density. Chlorophyll content index was negatively correlated with leaf dry matter content and leaf tissue density.

## Discussion

Environmental pollution is one of the main factors affecting the growth of urban plants. In fact, for the purpose of biological monitoring and bioremediation projects, the assessment of plant morphological and anatomical changes has been used to solve the selective tolerance of plant species to the affected environment. In recent years, with the aggravation of automobile exhaust, urban construction, sandstorm and other multiple pollution, atmospheric particulate pollution has become one of the major topics in urban environmental research. Therefore, a comprehensive understanding of the response of urban vegetation to dust pollution is of great significance for urban greening construction and allocation and coping with dust pollution.

Studies show that with the rapid development of spectral technology, the characteristics of high resolution, large amount of information, strong data continuity and simple access are also highlighted [38, 39]. For example, under the conditions of heavy metal pollution, pest and disease threat, drought stress, etc., its position often deviates to a certain extent, but the response law of the red edge position to dust pollution stress has not been verified at present. Generally speaking, there are two main directions for the shift of red edge position, one is the shift to long wave direction, that is, “red shift” phenomenon, the other is the shift to short wave direction, that is, “blue shift” phenomenon. The slope of red edge can reflect the chlorophyll content of plants and is often closely related to the photosynthetic rate of plants [38–43]. Therefore, the application of spectrum technology to dust pollution monitoring can achieve the purpose of obtaining air pollution information in a large scale, quickly and in real time. Data analysis shows that hyperspectral data can quickly predict the dust deposition on leaf surface of *L. quihoui* in real time, and a prediction model of dust deposition on leaf surface is established according to its reflection spectrum. Studies have shown that the slope of red edge has a good indication of chlorophyll content index. Therefore, we suspect that the cause of this phenomenon may be related to the influence of leaf covering on plant photosynthesis. In addition, the change trend of red edge position of *L. quihoui* before and after dust deposition was consistent in different urban environments. Compared with clean leaves, the red edge position of leaves after dust deposition has obvious “blue shift”. In different urban environments, the biggest moving distance of “blue shift” was the street, the next was the community, and the smallest was the park. This shows that the greater the dust deposition on leaves, the more severe the impact on the red edge position of leaves. To sum up, the slope and position of red edge were extremely sensitive to the disturbance of dust stagnation on leaf surface.

Some studies have shown that urban dust pollution leads to slight changes in leaf structure [36, 37] and this conclusion has been confirmed in our research. Different from previous studies, we chose the open urban environment as the experimental environment, which is more conducive to accurately explain the response of plants to dust pollution. In addition, we found that in the studied species, the observed changes in plant functional traits are relatively obvious. In different gradients of pollution, with the increase in dust deposition on the leaf surface, the specific leaf area and chlorophyll content index gradually decreased, and reached extremely significant differences in different environments. Specific leaf area can represent the adaptability of plants to the environment and the ability to obtain resources, which is closely related to the survival strategies of plants [16]. Plants with lower leaf area have stronger adaptability to arid environment with poor resources, while plants with higher leaf area have stronger ability to maintain nutrition in vivo [27, 38, 44, 45]. In this study, with the increase of dust deposition, the specific leaf area of plants decreased significantly. This shows that urban plants adapt to the influence of urban atmospheric particulate matter by reducing the specific leaf area. Our conclusion is consistent with the change of specific leaf area under high temperature and drought environment [46]. Chlorophyll content index can reflect the photosynthetic capacity of plant leaves [16]. In this study, with the increase of dust deposition, chlorophyll content index in plants decreased significantly. We suspect that this may be related to dust coverage. The leaves were covered by dust, the area of the leaves receiving light was greatly reduced. Leaf thickness plays a key role in determining the physical structure strength of plant leaves [47, 48]. In this study, with the increase of dust deposition, the leaf thickness increased significantly. This shows that in the environment polluted by dust, increasing the leaf thickness can avoid the mechanical damage to leaves caused by dust deposition on leaves. Leaf dry matter content can reflect the ability of plants to retain nutrients. Leaf tissue density is closely related to the defense ability and mechanical organization ability of plant leaves [49, 50]. In this study, with the increase of dust deposition on leaves, the dry matter content and tissue density of leaves increased significantly. This shows that plants use more resources for the construction of defense structures in order to reduce the damage degree of leaves.

There are various relationships among functional traits of species, the most common of which is trade-off [51]. This trade-off relationship is a combination of traits formed after natural selection, also called “ecological strategy”, that is, species are arranged in the most suitable or competitive position along a certain ecological strategy axis [19, 49, 52]. In this study, there was a significant positive correlation between specific leaf area and chlorophyll content index. Specific leaf area is related to drought tolerance of plants [48]. With the increase in dust deposition, specific leaf area decreases to reduce transpiration. At the same time, due to the decrease of specific leaf area, the leaf area of photosynthesis is also reduced, which leads to the decrease of chlorophyll content index. Chlorophyll content index was negatively correlated with leaf dry matter content and leaf tissue density. The leaf dry matter content can reflect the ability of plants to retain nutrients [53]. The leaf tissue density is closely related to the defense ability and mechanical organization ability of plant leaves [54, 55]. This shows that plants can slow down their growth by increasing the density of leaf tissues and the content of dry matter in leaves, reserve more carbon for defensive structures, and use more synthetic substances to increase the construction of protective tissues, so as to adapt to the environment polluted by urban atmospheric particulate matter. Among the limited resources, because plants use more resources and nutrition for the construction of leaf defense structure, the resources for photosynthesis are correspondingly reduced [56, 57]. This also reflects an “ecological strategy” for plants to cope with the adverse external environment.

## Conclusion

(1) The average amount of dust deposition per unit area of *L. quihoui* leaves was significantly different in different urban environments, and the overall performance was street > community > park. In different urban environments, the trend of leaf reflection spectrum curve tends to be consistent. There were five main reflection peaks and five main absorption valleys in *L. quihoui* in three different urban environments, and their positions and ranges were basically the same.

(2) The spectral reflectance of dust particles on the leaves of *L. quihoui* before and after removal was obviously different (clean leaves > dust-stagnant leaves). In the range of 695 ~ 1400 nm, the spectral reflectance of *L. quihoui* with or without dust was the most distinguished, which indicates that this band is the sensitive range of its spectral response. This characteristic has universal applicability in the environment. The dust deposition on leaves has significant influence on the slope and position of red edge. The slope of red edge was the largest in park environment, the second in community environment and the smallest in street environment. After dust deposition, the red edge position of the leaf surface has obvious “blue shift”, and the greater the dust deposition on the leaf surface, the more severe the influence on the red edge position of the leaf surface.

(3) The forecast model of dust deposition amount established by simple ratio index (y = 2.5171x + 0.3806, *R*^2^ = 0.7873, RMSE = 0.187) has an average accuracy of 99.98%.

(4) With the increase in dust deposition on the leaf surface, the specific leaf area and chlorophyll content index decreased gradually. The leaf dry matter content, leaf tissue density and leaf thickness increased gradually. In the environment polluted by dust, *L. quihoui* generally presents a combination of characters with low specific leaf area, low chlorophyll content index, high leaf dry matter content, high leaf tissue density and large leaf thickness, which reflects the trade-off strategy between investment and return of plant leaves in structure construction, and fully demonstrates that plants adjust their own functional traits in order to adapt to the adverse stress caused by the habitat characteristics of urban environmental pollution.

## Materials and methods

### Study area and sample collection

**Fig. 8:**
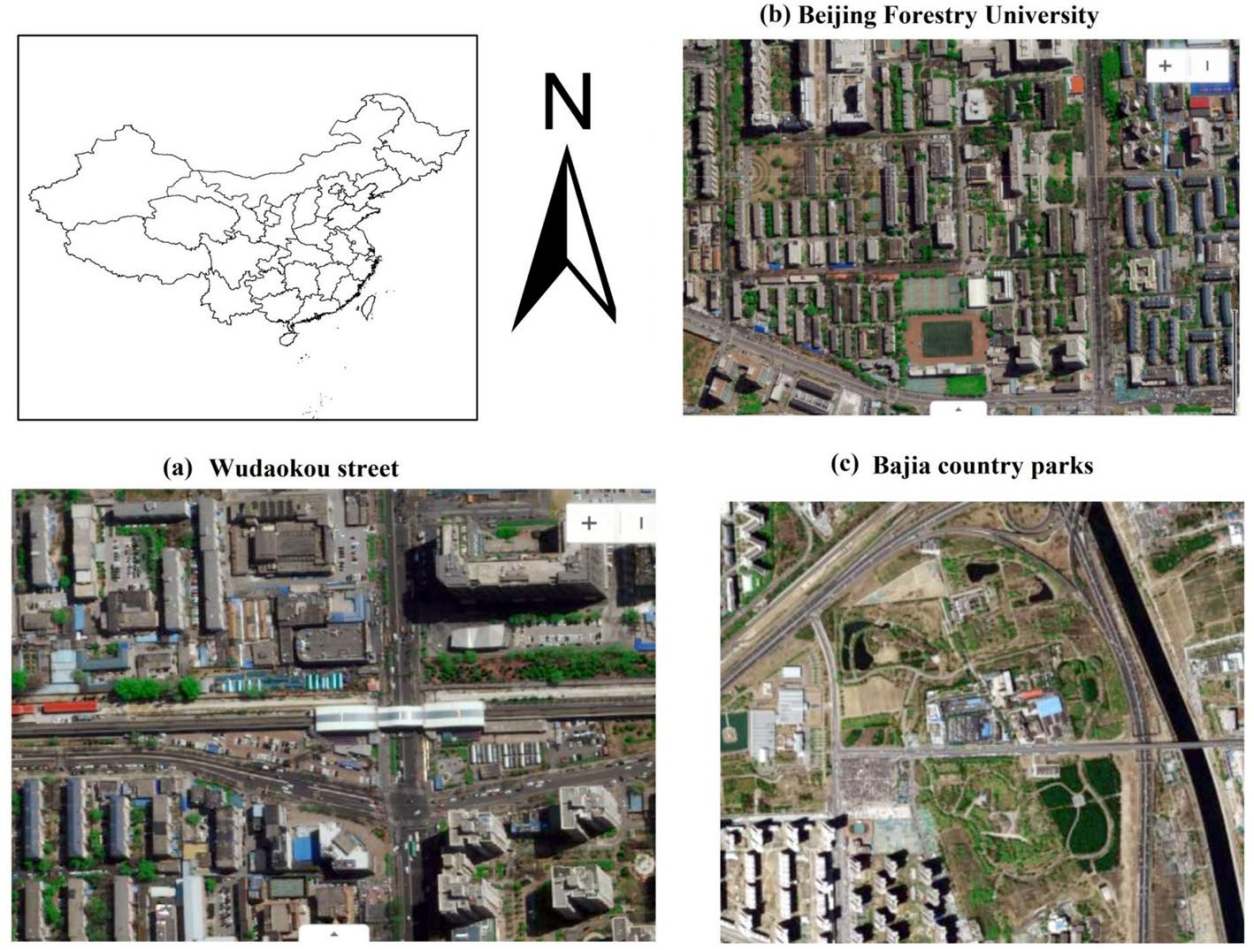
Study area and location of sampling. **a** Street environment, **b** Community environment, **c** Park environment. The map of Fig. 8a-c was downloaded from Google Maps (maps.google.com.hk). We only intercepted the study area and did not change any position in the map. The map of China is downloaded from the website of China Bureau of Surveying and Mapping (http://www.sbsm.gov.cn/), and the map is complete

Haidian District, located between the central city of Beijing and the ecological public welfare zone in the outer suburbs. It is an important urban function expansion area in Beijing, with a total area of 431 km^2^, accounting for 2.53% of the total area of Beijing, ranging from 116°03′—116°23 east longitude to 39°53 ′—40°09 ′ north latitude (Data from http: //www.weather.com.cn/). Haidian District is located in the warm temperate semi-humid and semi-arid continental monsoon climate zone, with an average annual precipitation of 483.5 mm and an average annual temperature of 12.3 °C. As shown in Fig. 8, the sampling areas are located in Wudaokou Street, Beijing Forestry University Campus and Bajia country parks, representing the highway environment (low density urban green area or high pollution area, T1), community environment (medium density urban green area or medium pollution area, T2) and park environment (high density urban green area or relatively clean area, T3). The results show that the parameters such as leaf surface coat density, leaf mass, leaf area and crown structure are important factors affecting the dust deposition benefits of plants. *Ligustrum quihoui* Carr. is a small shrub of Ligustrum in Oleaceae. In Beijing, it is often used as an important hedge material in landscaping. It has the characteristics of dense villi, slightly concave veins, abundant leaves and evergreen all the year round. It is mainly responsible for the decomposition, absorption and fixation of atmospheric particulate matter by vegetation in Beijing in winter.

In this study, the age of the studied tree species is 8 years. Leaf samples were collected from 10: 00 am to 12: 00 am in December, 2018. Thirty healthy *L. quihoui* seedlings were selected from three experimental sites, and the planting location was not covered by tall buildings and large trees. Three hundred sixty mature and healthy leaves were cut evenly in each test environment, put into a clean tray gently, and immediately brought back to the laboratory to measure related indexes. The linear distance between the sampling site and the experimental test site was about 10 km, and the time from in vitro to spectral collection of leaf samples was controlled within 30 min, thus ensuring that the leaf samples maintain their original growth activity. Under FlexSEM1000 electron scanning microscope (Hitachi, Osaka, Japan), it was observed that the shape of leaf particles was irregular block, sphere and polymer, and the particle size was less than 10 μm, in which the proportion of PM_10_ was 67.16%. Prof. Chengyang Xu was responsible for the identification of plant samples involved in this study (refer to Flora of China for identification information).

### Collection of leaf reflection spectrum and determination of dust deposition

**Fig. 9:**
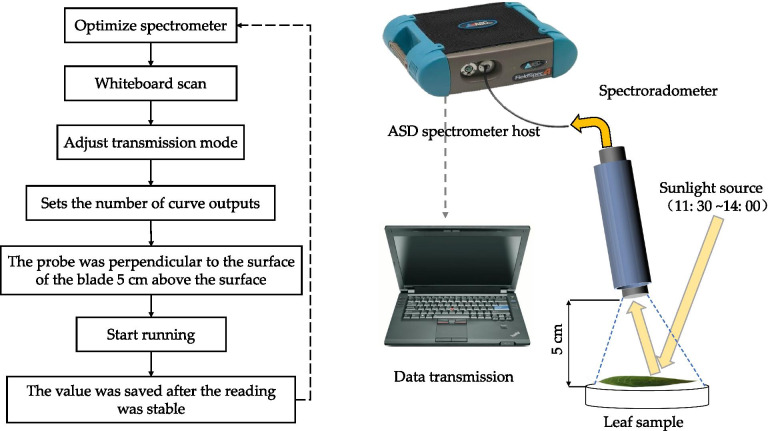
Flow chart of spectral measurement [58]

The experimental steps were as follows: first leaf mass measurement, first spectral data collection, second leaf mass measurement, second spectral data collection and leaf area measurement. Leaf spectrum was collected by ASD (Analytical Spectral Device, Almero, Netherlands) FieldSpec3 portable near infrared spectrometer, and the wavelength range of the instrument was 300 ~ 2500 nm. The resolution was 3 ~ 700 nm. Sampling interval were 1.4 nm (350 ~ 1000 nm) and 2 nm (1000 ~ 2500 nm). Angle of view was 30. Scanning time was 0.1 s, the spectral reflection curve was measured for 10 times, and the final output curve was automatically averaged. The spectrum acquisition processes were dark scanning, whiteboard scanning, transmission mode adjustment, instrument probe avoiding the center vein perpendicular to 4 cm above the sample center, and saving the value after reading was stable (Fig. 9).

According to the particle size, dust can be divided into floating dust, floating dust (D < 1 μm) and falling dust (D < 10 μm). There are three main movement modes (suspension, floating and desposition) [5]. Here, we mainly study the dust on the leaves. First, measure the initial mass of leaves with a balance of 1/10000, immerse them in deionized water, and scrub off the dust and impurities on the leaves with sanitary napkins. Then, clean absorbent paper will absorb the moisture on the leaves and measure the mass of leaves for the second time. The leaf area was measured by LI-3000C portable leaf area scanner (LI-COR, Boston, USA). Dust deposition per unit area was the ratio of leaf mass to leaf area before and after dust removal.

### Determination of leaf functional traits

The functional characteristics of plant leaves can intuitively reflect the response strategies of plants to environmental changes, among which specific leaf area, leaf thickness, leaf dry matter content, chlorophyll content index, leaf tissue density and other indicators directly affect the basic behavior, function and ecological strategy of plants, which are often considered as one of the best variables on the axis of plant utilization of environmental resources [59]. Therefore, in this study, we selected the above five functional traits. After spectrum collection, the leaf area (LA, mm^2^) was measured by LI-3000C leaf area scanner (LI-COR, Boston, USA). Chlorophyll content index (CCI) was measured by CCM-200 Plus portable chlorophyll meter (OPTI-Science, Tyngsboro, MA, USA). The leaf thickness (LT, mm) was measured by TM004 digital vernier caliper (Jinnuo, Nanjing, China). The fresh weight of leaves was measured by XPR5003S electronic balance (SAID, Shaoxing, China), then soaked in deionized water for 12 h, and the saturated fresh weight of leaves (LFW, g) was weighed. Finally, put all the leaves into an oven (65 °C) to dry to a constant mass, and weigh the dry mass of leaves (LDW, g).

1 $$\mathrm{SLA}=\mathrm{LA}/\mathrm{LDW},{\mathrm{cm}}^2/\mathrm{g}$$

2 $$\mathrm{LDMC}=\mathrm{LDW}/\mathrm{LFW},\mathrm{g}/\mathrm{g}$$

3 $$\mathrm{LTD}=\mathrm{LDW}/\left(\mathrm{LT}\times \mathrm{LA}\right),\mathrm{g}/{\mathrm{cm}}^3$$

### Data processing

Spectral data were preprocessed by using Viewspecpro software, and then analyzed by using Origin 2019b software. Linear regression model was selected as the prediction model of dust deposition on leaves, and spectral parameters were used as independent variables and dust deposition as dependent variables for linear fitting. Pearson correlation analysis was used to analyze the correlation between leaf functional traits. In this study, all drawings and tables were made in Origin 2019b software. The accuracy calculation formula of the prediction model was as follows:

4 $$\mathrm{Model}\ \mathrm{accuracy}\ \left(\%\right)=1-\frac{\left|w1-w2\right|}{100}\times 100\%$$

In the formula, w1 is the measured value and w2 is the predicted value.

## Data Availability

The data involved in the article were all shown in the figures and tables. However, there are still available from the first author on reasonable request.

## Abbreviations

SLA: Specific leaf area
LDMC: Leaf dry matter content
CCI: Chlorophyll content index
LTD: Leaf tissue density
LT: Leaf thickness
LFW: Leaf saturated fresh weight
LV: Leaf volume
LDW: Leaf dry weight
